# Ion channels as potential redox sensors in lysosomes

**DOI:** 10.1080/19336950.2019.1684428

**Published:** 2019-10-30

**Authors:** Jie Yu, Junsheng Yang

**Affiliations:** a Sports Science Research Center, Zhejiang College of Sports, Hangzhou, China; b Collaborative Innovation Center of Yangtze River Delta Region Green Pharmaceuticals, College of Pharmaceutical Sciences, Zhejiang University of Technology, Hangzhou, China

**Keywords:** Ion channels, lysosome, ROS, redox sensor

## Abstract

Lysosomes are central organelles that recycle materials and energy to maintain intracellular homeostasis. Lysosomes are capable of sensing environmental cues such as nutrition to regulate their function accordingly. Whether lysosomes can sense redox signaling, however, was unclear. Here in this review, we summarized recent evidence of lysosomal ion channel as redox sensors for this organelle. We also discussed their roles in lysosomal diseases that features imbalanced redox.

## Introduction

Lysosomes are membrane-bound acidic organelles that serve as “recycle centers” in almost all animal cells. Equipped with over 60 hydrolases, lysosomes are capable of degrading macromolecules including proteins, polysaccharides, and lipids to their building units for material re-use and energy homeostasis [1]. Lysosomes can fuse with “cargo” vesicles to receive substrates from both intracellular sources (autophagy via autophagosomes) and extracellular sources (endocytosis and phagocytosis via endosomes and phagosomes) [2] and can transport products of degradation (e.g. amino acids, fatty acids and monosaccharides) out of lysosomes by transporters or membrane trafficking [3].

Besides luminal hydrolases, lysosomes also harbor more than 50 types of proteins on their limiting membranes, with a number of them confirmed as transporters and ion channels. A still expanding list includes: V-ATPase as the proton pump that acidify lysosome lumen; NPC1 that transport cholesterol; SLC38A9 that senses and transports arginine; and TRPMLs, TPCs, BK, TMEM175, P2X4, CLCs as ion channels. These proteins play crucial roles in maintaining lysosomal homeostasis which is optimized for hydrolases activities, transporter activities and membrane trafficking [1]. Moreover, the recently revealed mechanisms of how mTORC1 docks on lysosome surface and then regulates the balance of cellular growth and autophagy suggests that lysosomes are far more than merely cellular waste bags, but rather key players in intracellular nutrient and energy sensing and signaling [4,5].

Reactive oxygen species (ROS) were first considered only as harmful chemical species that causes oxidative damage to molecules such as DNA, proteins, and lipids in cells, but were later on also considered to work as second messengers in redox signaling that can regulate a variety of biological processes [6–8]. In line with this notion, evidence suggested that autophagy can be regulated by ROS [9]. Whether lysosomes can be directly affected by ROS in autophagy regulation was however unclear. Also, whether and how lysosomes’ other diverse functions (e.g. exocytosis, vesicular trafficking, Ca^2+^ signaling) can be regulated by intracellular redox signals remain unclear open questions.

When considering the identity of redox sensors on organelles like lysosomes, it is natural to think about membrane proteins. Among them, ion channels are particularly “suspicious” because they can undergo redox modulations and can be activated/deactivated, thus are capable of sensing diverse stimuli including chemical signals. A number of ion channels have actually been identified as redox sensors on plasma membrane or other organelles [10–15].

However, studying lysosomal ion channels could be technically challenging because of lysosomes’ small sizes (100–500 nm in diameter), dynamic motility as well as their active fusion and fission [1]. Fortunately, with the development of techniques such as whole-lysosome patch-clamp [16] and planar patch clamp [17], together with lysosome-specific immunofluorescence, it is now possible to record current through lysosome memebranes, therefore, made it possible to characterize lysosomal ion channels under various conditions.

Here in this review, we will summarize recent findings that indicate one ion channel, TRPML1, as the first redox sensor on lysosomes that mediate ROS-induced autophagy and also discuss other lysosomal ion channels that might potentially respond to redox signaling.

## The first identified redox sensor on lysosomes: TRPML1

Transient receptor potential mucolipin 1 (TRPML1), encoded by the *MCOLN1* gene, is an ion channel that belongs to the mammalian mucolipin subfamily of the transient receptor potential (TRP) superfamily of cation channels [18]. TRPML1 is a Ca^2+^-permeable, nonselective cation channel that is mainly localized on late endosomes and lysosomes (LELs). Loss of function mutations of human TRPML1 leads to a lysosomal storage disease (LSD) named Mucolipidosis type IV disease (ML-IV), manifesting lysosomal storage and deficiency in lysosomal trafficking [18–20]. Studies during the past decade have revealed that TRPML1 serves as the primary Ca^2+^ release channel on lysosomes and is a key player in regulating multifaceted lysosomal functions including lysosomal exocytosis [21–23], nutrient sensing [24], lysosome motility [25], autophagy [26] and of most interest to this review, oxidative stress sensing [27].

In a study by Zhang et al. [27], the authors reported for the first time that lysosomal TRPML1 current can be activated directly by oxidants such as hydrogen peroxide and chloramine T, or chemicals such as carbonyl cyanide m-chlorophenylhydrazone (CCCP) that can induce mitochondrial ROS production. This activation of TRPML1 by oxidants or ROS is missing in TRPML1 knockout cells and also can be blocked by antioxidants or TRPML1-specific antagonists, suggesting its specificity. Moreover, the TRPML1 current induced by oxidants is quick on lysosome patch-clamps, indicating that it is a direct activation. The authors further studied the consequences of the redox activation of TRPML1 and found that transcription factor EB (TFEB), the master regulator of lysosome biogenesis and autophagy is activated via TRPML1-mediated Ca^2+^ signaling. Not surprisingly, lysosome biogenesis and autophagy levels are both elevated upon TRPML1 activation by oxidants or CCCP treatment. Very interestingly, TRPML1 is not required for autophagy induced by mTOR inhibition, suggestive of a redox-specific role-played by this channel in autophagy regulation. Also, TRPML1 is required for the removal of excessive ROS induced by CCCP, presumably through mitophagy, a specialized autophagy that removes damaged mitochondria.

## Redox-sensing ion channels on lysosomes of certain cells: TRPM2

Besides ion channels that are located on lysosomes in all cell types, some ion channels are localized on lysosomes only in certain cell types. Among them, transient receptor potential melastatin 2 (TRPM2) is one that is most extensively studied.

TRPM2 is a Ca^2+^-permeable nonselective cation channel that belongs to the melastatin subfamily of TRP superfamily [28,29]. Human TRPM2 is expressed in various tissues, with especially high levels in brain and peripheral blood cells [30]. TRPM2 are localized on plasma membrane in most cell types, and can be activated by a variety of stimuli including ADP-ribose (ADPR), ADPR-2ʹ-phosphate (ADPRP), Ca^2+^, heat and oxidative stress [28,29,31]. While it is known that TRPM2 activation leads to Ca^2+^ entry to cells and is linked to diseases like ischemia-reperfusion injury and bipolar disorder, the physiological function of TRPM2 is controversial [25,28,32,33]. Very interestingly, TRPM2 is also found on lysosomal membranes of pancreatic β cells and mediates oxidative stress-induced β cell death, as well as on lysosomal membranes of dendritic cells and mediates cell maturation and chemotaxis [34,35]. Moreover, recent evidence suggests TRPM2 can also reside on lysosomal membranes in cancer cells and modulate their migration by regulating actin remodeling [36]. Plus, oxidative-stress-induced activation of lysosomal TRPM2 not only triggers the release of Ca^2+^, but also Zn^2+^ from lysosomes [36,37]. These findings suggest that TRPM2-mediated cytosolic Zn^2+^ elevation is also closely involved in pancreatic β cells death and actin cytoskeleton remodeling, although the identity of the responsible Zn^2+^ transporter is still unclear. Altogether, lysosomal TRPM2 appears to play complex physiological functions associated with specific cell types.

## Other possible redox-sensing lysosomal ion channels

In addition to the channels discussed above, there still might be more redox-sensing ion channels on lysosomes. For example, large conductance Ca^2+^-activated K^+^ channels (also known as BK for Big Potassium, Maxi-K, or Slo1), first found on plasma membranes, are reported to be modulated by oxidizing and reducing reagents {DiChiara, 1997 #241}. Accumulating evidence suggests that cysteines and methionines on BK undergo complex oxidation, which consequently affect their channel property [for a detailed review, see 13]. BK channel on mitochondria (mitoBK) is a crucial regulator of mitochondrial function and ROS generation [38–41]. In recent studies, BK is discovered to also be a lysosomal potassium channel that facilitates TRPML1 functions and lysosomal Ca^2+^ store maintenance [42,43]. Whether lysosomal BK also undergoes same oxidation modulation would be an interesting question to explore. TMEM175, another lysosomal potassium channel, has been reported to affect lysosome functions, mitochondrial respiration, and pathogenesis of Parkinson’s disease [44,45]. It is still unclear whether TMEM175 is directly involved in redox sensing. Chloride channel CLC-3 has been reported to be a lysosomal channel [46] and can mediate ROS signaling [47]. A recent study also indicated that CLC-3 current can be activated by ROS induced by zoledronic acid (ZA) [48]. Although it is now more accepted that CLC-3 is localized predominantly on early and late endosomes while CLC-7 is the major chloride channel on lysosomes [1,49,50], it would be interesting to check whether CLC-7 shows similar redox-sensitivity.

## Redox-sensing lysosomal ion channels and diseases

Elevated ROS is a common feature of many LSDs [51,52]. Interestingly, several of them are linked with compromised TRPML1 functions. Fibroblasts from ML-IV patients have constitutively higher ROS when compared to fibroblasts from normal persons [27]. In line with this, knockdown of TRPML1 in retinal pigmented epithelial 1 (RPE1) cells leads to mitochondria fragmentation and ROS buildup [53]. In Niemann–Pick Disease Type C (NPC), another LSD, associated with defected NPC1 or NPC2 proteins and the consequent lysosomal accumulation of lipids such as cholesterol, TRPML1 channel activity is reduced by 70% [54] while ROS level is elevated when compared with WT fibroblasts [27,55]. Similarly, Fabry disease, an X-linked LSD caused by alpha galactosidase (GLA) deficiency and the accumulation of its substrates such as globotriaosylceramide (Gb3), also manifest decreased TRPML1 activity [56] and abnormal redox status, including increased ROS generation in cultured Fabry endothelial cells [57] as well as increased oxidative damage to macromolecules in Fabry patients [58]. Altogether, these data indicate that defected TRPML1 activity might be a common feature for different LSDs. Considering TRPML1’s role in removal of excessive ROS, whether restoring TRPML1 genetically or pharmaceutically can alleviate the abnormal oxidative stress and improve cells’ healthiness in these diseases will be tempting to test. It is also worth exploring whether other LSDs with similar redox imbalance also features similar TRPML1 function defects.

Besides LSDs, emerging evidence suggests that TRPML1 is also important for cancer cell survival [59–61]. Interestingly, the effect of TRPML1 activity modulation on cancer cell survival differs, possibly dependent on the type of cancers and stimuli. Specifically, TRPML1 inhibition suppresses the growth of triple-negative breast cancers (TNBCs) [59] and reduces the proliferation of oncogenic *HRAS*-expressing cancer cells [61]. On the other hand, a very recent research reported that glioblastoma (GBM) patients survival are negatively correlated with TRPML1 expression level, while glioma cells are vulnerable to ROS when TRPML1 is activated [60].

As mentioned earlier in this review, although TRPM2 has been linked to multiple diseases, lysosomal TRPM2 appears to show specialized functions in immune cells. Besides β cells and dendritic cells, TRPM2 has also been reported to localize on lysosomes in peritoneal macrophages in a recent study [62]. Interestingly, TRPM2 knockout leads to decreased phagosome-lysosome fusion in macrophages, resulting disrupted phagosome acidification and bacterial killing. In line with this, TRPM2 knockout mice also appeared to have impaired clearance of invading bacteria and thus sepsis. This new finding provided a hint that lysosomal TRPM2 might facilitate the fusion of lysosome with other organelles to fulfill their responsible functions.

## Concluding remarks

**Figure 1.**
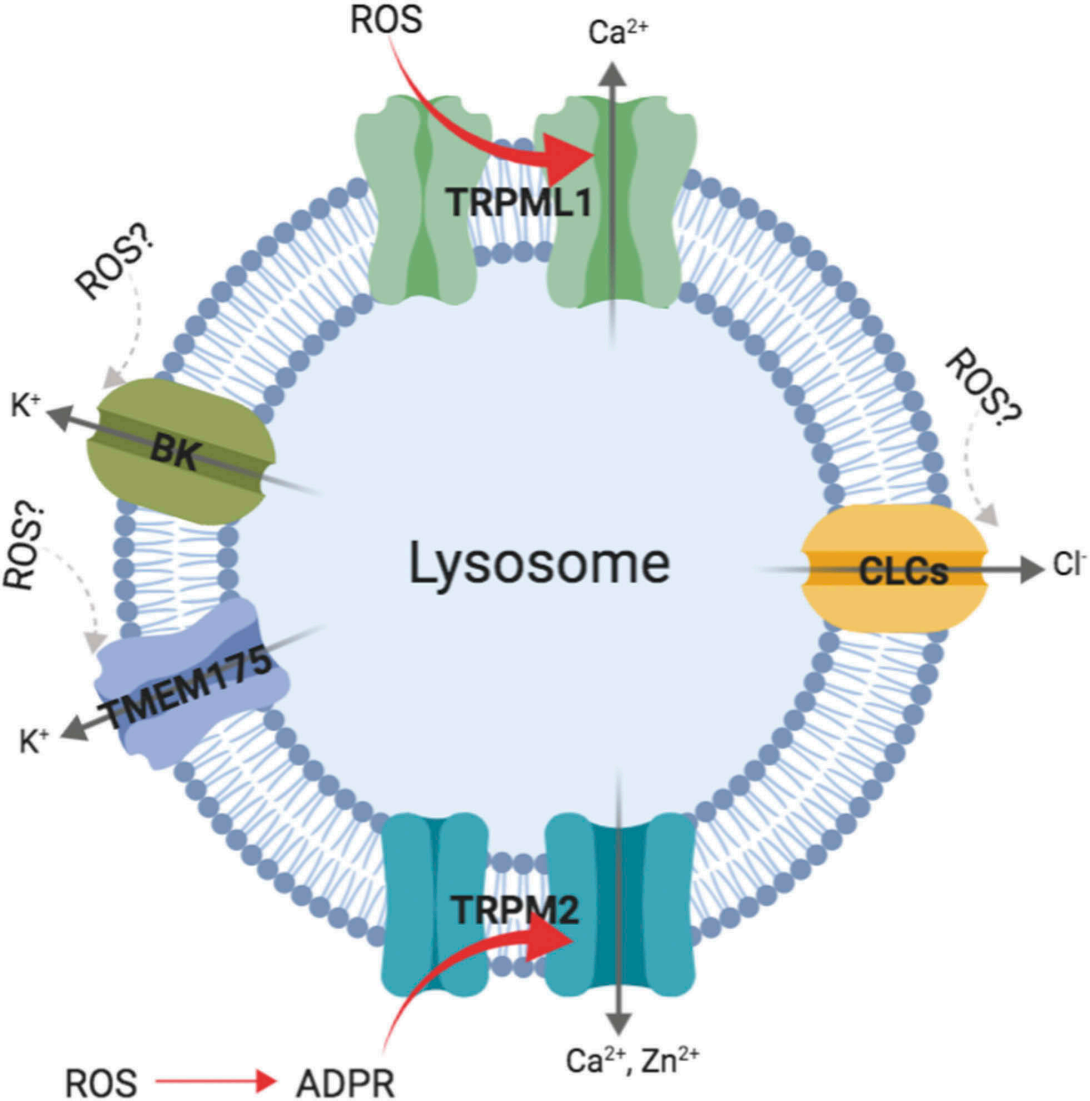
Ion channels as potential redox sensors in lysosomes. A schematic summary of the lysosomal ion channels as potential redox sensors reviewed in this article. TRPML1 can be activated by intracellular ROS, leading to Ca^2+^ release, followed by TFEB activation and autophagy boost. TRPM2, found on the lysosomes of immune cells, are indirectly activated by ROS via ADPR, resulting an increase of intracellular Ca^2+^ and Zn^2+^, which are closely involved in β cell death and actin remodeling. BK channels on plasma membrane undergo complex oxidation modulation, whether lysosomal BK can be regulated by the same mechanism is unclear. Similarly, more work is needed for the role of redox status on other lysosomal channels such as TMEM175 and CLCs.

Sensing redox cues in the environment is not only a task cells need to handle but also challenges to intracellular organelles. Lysosomes, as key organelle that coordinate intracellular homeostasis, are no exceptions to these challenges. Here we summarized recent discoveries that indicate lysosomal ion channels as potential redox sensors (for a summary, see Figure 1).

The identification of TRPML1 as lysosomal redox sensor filled a long missing link between ROS and autophagy thus is of high significance. Also, this finding easily raises more follow-up questions. Cell biology-wise, whether and how do ROS affect other TRPML1-mediated lysosome functions (such as lysosome motility, lysosome tabulation, and reformation, lysosomal exocytosis and membrane trafficking)? Does lysosome respond to different levels of ROS differently? Physiology-wise, would the correction of elevated ROS in TRPML1 k.o. cells alleviate symptoms of diseases caused by TRPML1 deficiency (e.g. ML-IV, NPC)? Mechanism-wise, how exactly does TRPML1 get activated by redox signals? With the newly solved structures of TRPML1 [63–65], more details on the biochemical and structural changes of TRPML1 upon redox-regulation can be expected. Similarly, the latest discovery of zebrafish TRPM2’s structures at resting and active states has also shed light on possible mechanisms how this channel is activated/sensitized by oxidative signals [66].

Whether there are more lysosomal ion channel redox sensors? To answer this question, on the one hand, knowledge of the identity and channel property of more lysosomal channels will be needed; on the other hand, ion channels that function on lysosome only in specialized cells (such as TRPM2, TRPA1) add another level of complexity to the question and will require more thorough understanding of cell type-specific channel distribution and functions.

Altogether, researches on lysosomal ion channels’ redox-sensing properties are only in its early stage, but could be of great value. The accumulation of structural information of these channels, together with the findings of more channel-specific agonists/antagonists, could make them a group of potential drug targets for disorders that features disrupted redox signaling.

## References

[1] Xu H, Ren D. Lysosomal physiology. Annu Rev Physiol. 2015;77:57–80. 25668017 10.1146/annurev-physiol-021014-071649 PMC4524569

[2] Luzio JP, Pryor PR, Bright NA Lysosomes: fusion and function. Nat Rev Mol Cell Biol. 2007;8(8):622–632. 17637737 10.1038/nrm2217

[3] Saftig P, Klumperman J Lysosome biogenesis and lysosomal membrane proteins: trafficking meets function. Nat Rev Mol Cell Biol. 2009;10(9):623–635. 19672277 10.1038/nrm2745

[4] Settembre C, Fraldi A, Medina DL, et al Signals from the lysosome: a control centre for cellular clearance and energy metabolism. Nat Rev Mol Cell Biol. 2013;14(5):283–296. 23609508 10.1038/nrm3565 PMC4387238

[5] Appelqvist H, Waster P, Kagedal K, et al The lysosome: from waste bag to potential therapeutic target. J Mol Cell Biol. 2013;5(4):214–226. 23918283 10.1093/jmcb/mjt022

[6] Schieber M, Chandel NS ROS function in redox signaling and oxidative stress. Curr Biol. 2014;24(10):R453–462. 24845678 10.1016/j.cub.2014.03.034 PMC4055301

[7] D’Autreaux B, Toledano MB ROS as signalling molecules: mechanisms that generate specificity in ROS homeostasis. Nat Rev Mol Cell Biol. 2007;8(10):813–824. 17848967 10.1038/nrm2256

[8] Finkel T Signal transduction by reactive oxygen species. J Cell Biol. 2011;194(1):7–15. 21746850 10.1083/jcb.201102095 PMC3135394

[9] Scherz-Shouval R, Elazar Z Regulation of autophagy by ROS: physiology and pathology. Trends Biochem Sci. 2011;36(1):30–38. 20728362 10.1016/j.tibs.2010.07.007

[10] Shimizu S, Takahashi N, Mori Y TRPs as chemosensors (ROS, RNS, RCS, gasotransmitters). Handb Exp Pharmacol. 2014;223:767–794. 24961969 10.1007/978-3-319-05161-1_3

[11] Dong Z, Shanmughapriya S, Tomar D, et al Mitochondrial Ca(2+) uniporter is a mitochondrial luminal redox sensor that augments MCU channel activity. Mol Cell. 2017;65(6):1014–1028 e1017. 28262504 10.1016/j.molcel.2017.01.032 PMC5357178

[12] Ogawa N, Kurokawa T, Mori Y Sensing of redox status by TRP channels. Cell Calcium. 2016;60(2):115–122. 26969190 10.1016/j.ceca.2016.02.009

[13] Sahoo N, Hoshi T, Heinemann SH Oxidative modulation of voltage-gated potassium channels. Antioxid Redox Signal. 2014;21(6):933–952. 24040918 10.1089/ars.2013.5614 PMC4116129

[14] Peers C, Boyle JP Oxidative modulation of K+ channels in the central nervous system in neurodegenerative diseases and aging. Antioxid Redox Signal. 2015;22(6):505–521. 25333910 10.1089/ars.2014.6007

[15] Naziroglu M, Cig B, Ozgul C Modulation of oxidative stress and Ca(2+) mobilization through TRPM2 channels in rat dorsal root ganglion neuron by hypericum perforatum. Neuroscience. 2014;263:27–35. 24434769 10.1016/j.neuroscience.2014.01.006

[16] Dong X. P, Cheng X, Mills E, et al The type IV mucolipidosis-associated protein TRPML1 is an endolysosomal iron release channel. Nature. 2008;455(7215):992–996. 18794901 10.1038/nature07311 PMC4301259

[17] Schieder M, Rotzer K, Bruggemann A, et al Planar patch clamp approach to characterize ionic currents from intact lysosomes. Sci Signal. 2010;3(151):pl3. 21139138 10.1126/scisignal.3151pl3

[18] Wang W, Zhang X, Gao Q, et al TRPML1: an ion channel in the lysosome. Handb Exp Pharmacol. 2014;222:631–645. 24756723 10.1007/978-3-642-54215-2_24

[19] Venkatachalam K, Wong C. O, Zhu MX The role of TRPMLs in endolysosomal trafficking and function. Cell Calcium. 2015;58(1):48–56. 25465891 10.1016/j.ceca.2014.10.008 PMC4412768

[20] Nilius B, Owsianik G, Voets T, et al Transient receptor potential cation channels in disease. Physiol Rev. 2007;87(1):165–217. 17237345 10.1152/physrev.00021.2006

[21] Medina DL, Fraldi A, Bouche V, et al Transcriptional activation of lysosomal exocytosis promotes cellular clearance. Dev Cell. 2011;21(3):421–430. 21889421 10.1016/j.devcel.2011.07.016 PMC3173716

[22] Samie M, Wang X, Zhang X, et al A TRP channel in the lysosome regulates large particle phagocytosis via focal exocytosis. Dev Cell. 2013;26(5):511–524. 23993788 10.1016/j.devcel.2013.08.003 PMC3794471

[23] Park S, Ahuja M, Kim MS, et al Fusion of lysosomes with secretory organelles leads to uncontrolled exocytosis in the lysosomal storage disease mucolipidosis type IV. EMBO Rep. 2016;17(2):266–278. 26682800 10.15252/embr.201541542 PMC5290820

[24] Wang W, Gao Q, Yang M, et al Up-regulation of lysosomal TRPML1 channels is essential for lysosomal adaptation to nutrient starvation. Proc Natl Acad Sci U S A. 2015;112(11):E1373–1381. 25733853 10.1073/pnas.1419669112 PMC4371935

[25] Zhan K. Y, Yu P. L, Liu C. H, et al Detrimental or beneficial: the role of TRPM2 in ischemia/reperfusion injury. Acta Pharmacol Sin. 2016;37(1):4–12. 26725732 10.1038/aps.2015.141 PMC4722978

[26] Medina DL, Di Paola S, Peluso I, et al Lysosomal calcium signalling regulates autophagy through calcineurin and TFEB. Nat Cell Biol. 2015;17(3):288–299. 25720963 10.1038/ncb3114 PMC4801004

[27] Zhang X, Cheng X, Yu L, et al MCOLN1 is a ROS sensor in lysosomes that regulates autophagy. Nat Commun. 2016;7:12109. 27357649 10.1038/ncomms12109 PMC4931332

[28] Cheung JY, Miller BA Transient receptor potential-melastatin channel family member 2: friend or foe. Trans Am Clin Climatol Assoc. 2017;128:308–329. 28790515 PMC5525431

[29] Ramsey IS, Delling M, Clapham DE An introduction to TRP channels. Annu Rev Physiol. 2006;68:619–647. 16460286 10.1146/annurev.physiol.68.040204.100431

[30] Wu L. J, Sweet T. B, Clapham DE International union of basic and clinical pharmacology. LXXVI. Current progress in the mammalian TRP ion channel family. Pharmacol Rev. 2010;62(3):381–404. 20716668 10.1124/pr.110.002725 PMC2964900

[31] Kashio M, Tominaga M The TRPM2 channel: a thermo-sensitive metabolic sensor. Channels (Austin). 2017;11(5):426–433. 28633002 10.1080/19336950.2017.1344801 PMC5626354

[32] Sita G, Hrelia P, Graziosi A, et al TRPM2 in the brain: role in health and disease. Cells. 2018;7(7):82. 10.3390/cells7070082 PMC6070997 30037128

[33] Turlova E, Feng Z. P, Sun H. S The role of TRPM2 channels in neurons, glial cells and the blood-brain barrier in cerebral ischemia and hypoxia. Acta Pharmacol Sin. 2018;39(5):713–721. 29542681 10.1038/aps.2017.194 PMC5943904

[34] Lange I, Yamamoto S, Partida-Sanchez S, et al TRPM2 functions as a lysosomal Ca2+-release channel in beta cells. Sci Signal. 2009;2(71):ra23. 19454650 10.1126/scisignal.2000278 PMC2779714

[35] Sumoza-Toledo A, Lange I, Cortado H, et al Dendritic cell maturation and chemotaxis is regulated by TRPM2-mediated lysosomal Ca2+ release. FASEB J. 2011;25(10):3529–3542. 21753080 10.1096/fj.10-178483 PMC3177582

[36] Li F, Abuarab N, Sivaprasadarao A Reciprocal regulation of actin cytoskeleton remodelling and cell migration by Ca2+ and Zn2+: role of TRPM2 channels. J Cell Sci. 2016;129(10):2016–2029. 27068538 10.1242/jcs.179796

[37] Manna PT, Munsey TS, Abuarab N, et al TRPM2-mediated intracellular Zn2+ release triggers pancreatic beta-cell death. Biochem J. 2015;466(3):537–546. 25562606 10.1042/BJ20140747

[38] Malinska D, Mirandola SR, Kunz WS Mitochondrial potassium channels and reactive oxygen species. FEBS Lett. 2010;584(10):2043–2048. 20080090 10.1016/j.febslet.2010.01.013

[39] Ryu J,OU,SY, Jhun BS, Hurst S, et al Mitochondrial ion channels/transporters as sensors and regulators of cellular redox signaling. Antioxid Redox Signal. 2014;21(6):987–1006. 24180309 10.1089/ars.2013.5681 PMC4116125

[40] Sesti F Oxidation of K(+) channels in aging and neurodegeneration. Aging Dis. 2016;7(2):130–135. 27114846 10.14336/AD.2015.0901 PMC4809605

[41] Yang J, Yan X Oxidation of potassium channels in neurodegenerative diseases: a mini-review. CNS Neurol Disord Drug Targets. 2018;17(4):267–271. 29422009 10.2174/1871527317666180202110056

[42] Cao Q, Zhong XZ, Zou Y, et al BK channels alleviate lysosomal storage diseases by providing positive feedback regulation of lysosomal Ca2+ release. Dev Cell. 2015;33(4):427–441. 25982675 10.1016/j.devcel.2015.04.010

[43] Wang W, Zhang X, Gao Q, et al A voltage-dependent K(+) channel in the lysosome is required for refilling lysosomal Ca(2+) stores. J Cell Biol. 2017;216(6):1715–1730. 28468834 10.1083/jcb.201612123 PMC5461029

[44] Cang C, Aranda K, Seo Y. J, et al TMEM175 is an organelle K(+) channel regulating lysosomal function. Cell. 2015;162(5):1101–1112. 26317472 10.1016/j.cell.2015.08.002

[45] Jinn S, Drolet RE, Cramer PE, et al TMEM175 deficiency impairs lysosomal and mitochondrial function and increases alpha-synuclein aggregation. Proc Natl Acad Sci U S A. 2017;114(9):2389–2394. 28193887 10.1073/pnas.1616332114 PMC5338534

[46] Li X, Wang T, Zhao Z, et al The ClC-3 chloride channel promotes acidification of lysosomes in CHO-K1 and Huh-7 cells. Am J Physiol Cell Physiol. 2002;282(6):C1483–1491. 11997263 10.1152/ajpcell.00504.2001

[47] Fisher AB Redox signaling across cell membranes. Antioxid Redox Signal. 2009;11(6):1349–1356. 19061438 10.1089/ars.2008.2378 PMC2842114

[48] Wang L, Gao H, Yang X, et al The apoptotic effect of zoledronic acid on the nasopharyngeal carcinoma cells via ROS mediated chloride channel activation. Clin Exp Pharmacol Physiol. 2018;45(10):1019–1027. 29884989 10.1111/1440-1681.12979

[49] Xu H, Martinoia E, Szabo I Organellar channels and transporters. Cell Calcium. 2015;58(1):1–10. 25795199 10.1016/j.ceca.2015.02.006 PMC4458415

[50] Suzuki T, Rai T, Hayama A, et al Intracellular localization of ClC chloride channels and their ability to form hetero-oligomers. J Cell Physiol. 2006;206(3):792–798. 16222710 10.1002/jcp.20516

[51] Wei H, Kim SJ, Zhang Z, et al ER and oxidative stresses are common mediators of apoptosis in both neurodegenerative and non-neurodegenerative lysosomal storage disorders and are alleviated by chemical chaperones. Hum Mol Genet. 2008;17(4):469–477. 17989065 10.1093/hmg/ddm324

[52] Vitner EB, Platt FM, Futerman AH Common and uncommon pathogenic cascades in lysosomal storage diseases. J Biol Chem. 2010;285(27):20423–20427. 20430897 10.1074/jbc.R110.134452 PMC2898325

[53] Coblentz J, St Croix C, Kiselyov K Loss of TRPML1 promotes production of reactive oxygen species: is oxidative damage a factor in mucolipidosis type IV? Biochem J. 2014;457(2):361–368. 24192042 10.1042/BJ20130647

[54] Shen D, Wang X, Li X, et al Lipid storage disorders block lysosomal trafficking by inhibiting a TRP channel and lysosomal calcium release. Nat Commun. 2012;3:731. 22415822 10.1038/ncomms1735 PMC3347486

[55] Zampieri S, Mellon SH, Butters TD, et al Oxidative stress in NPC1 deficient cells: protective effect of allopregnanolone. J Cell Mol Med. 2009;13(9B):3786–3796. 18774957 10.1111/j.1582-4934.2008.00493.x PMC2832077

[56] Zhong XZ, Sun X, Cao Q, et al BK channel agonist represents a potential therapeutic approach for lysosomal storage diseases. Sci Rep. 2016;6:33684. 27670435 10.1038/srep33684 PMC5037385

[57] Shen J. S, Meng X. L, Moore DF, et al Globotriaosylceramide induces oxidative stress and up-regulates cell adhesion molecule expression in Fabry disease endothelial cells. Mol Genet Metab. 2008;95(3):163–168. 18707907 10.1016/j.ymgme.2008.06.016 PMC2593623

[58] Biancini GB, Vanzin CS, Rodrigues DB, et al Globotriaosylceramide is correlated with oxidative stress and inflammation in Fabry patients treated with enzyme replacement therapy. Biochim Biophys Acta. 2012;1822(2):226–232. 22085605 10.1016/j.bbadis.2011.11.001

[59] Xu M, Almasi S, Yang Y, et al The lysosomal TRPML1 channel regulates triple negative breast cancer development by promoting mTORC1 and purinergic signaling pathways. Cell Calcium. 2019;79:80–88. 30889511 10.1016/j.ceca.2019.02.010 PMC6698368

[60] Morelli MB, Amantini C, Tomassoni D, et al Transient receptor potential mucolipin-1 channels in glioblastoma: role in patient’s survival. Cancers (Basel). 2019;11(4):525. 10.3390/cancers11040525 PMC6521337 31013784

[61] Jung J, Cho KJ, Naji AK, et al HRAS-driven cancer cells are vulnerable to TRPML1 inhibition. EMBO Rep. 2019;20(4).pii:e46685 10.15252/embr.201846685 PMC6446245 30787043

[62] Zhang Z, Cui P, Zhang K, et al Transient receptor potential melastatin 2 regulates phagosome maturation and is required for bacterial clearance in escherichia coli sepsis. Anesthesiology. 2017;126(1):128–139. 27792045 10.1097/ALN.0000000000001430

[63] Chen Q, She J, Zeng W, et al Structure of mammalian endolysosomal TRPML1 channel in nanodiscs. Nature. 2017;550(7676):415–418. 29019981 10.1038/nature24035 PMC5901962

[64] Schmiege P, Fine M, Blobel G, et al Human TRPML1 channel structures in open and closed conformations. Nature. 2017;550(7676):366–370. 29019983 10.1038/nature24036 PMC5920536

[65] Zhang S, Li N, Zeng W, et al Cryo-EM structures of the mammalian endo-lysosomal TRPML1 channel elucidate the combined regulation mechanism. Protein Cell. 2017;8(11):834–847. 28936784 10.1007/s13238-017-0476-5 PMC5676595

[66] Huang Y, Winkler PA, Sun W, et al Architecture of the TRPM2 channel and its activation mechanism by ADP-ribose and calcium. Nature. 2018;562:145–149. 10.1038/s41586-018-0558-4 30250252

